# Sophoridine suppresses lenvatinib‐resistant hepatocellular carcinoma growth by inhibiting RAS/MEK/ERK axis via decreasing VEGFR2 expression

**DOI:** 10.1111/jcmm.16108

**Published:** 2020-11-18

**Authors:** Zhongwei Zhao, Dengke Zhang, Fazong Wu, Jianfei Tu, Jingjing Song, Min Xu, Jiansong Ji

**Affiliations:** ^1^ Key Laboratory of Imaging Diagnosis and Minimally Invasive Intervention Research the Fifth Affiliated Hospital of Wenzhou Medical University /Affiliated Lishui Hospital of Zhejiang University/ The Central Hospital of Zhejiang Lishui Lishui China; ^2^ Department of Radiology the Fifth Affiliated Hospital of Wenzhou Medical University /Affiliated Lishui Hospital of Zhejiang University/ The Central Hospital of Zhejiang Lishui Lishui China

**Keywords:** hepatocellular carcinoma, lenvatinib resistance, sophoridine, VEGFR2

## Abstract

Hepatocellular carcinoma (HCC) is one of the most lethal cancer types with insufficient approved therapies, among which lenvatinib is a newly approved multi‐targeted tyrosine kinase inhibitor for frontline advanced HCC treatment. However, resistance to lenvatinib has been reported in HCC treatment recently, which limits the clinical benefits of lenvatinib. This study aims to investigate the underlying mechanism of lenvatinib resistance and explore the potential drug to improve the treatment for lenvatinib‐resistant (LR) HCC. Here, we developed two human LR HCC cell lines by culturing with long‐term exposure to lenvatinib. Results showed that the vascular endothelial growth factor receptors (VEGFR)2 expression and its downstream RAS/MEK/ERK signalling were obviously up‐regulated in LR HCC cells, whereas the expression of VEGFR1, VEGFR3, FGFR1‐4 and PDGFRα/β showed no difference. Furthermore, ETS‐1 was identified to be responsible for VEGFR2 mediated lenvatinib resistance. The cell models were further used to explore the potential strategies for restoration of sensitivity of lenvatinib. Sophoridine, an alkaloid extraction, inhibited the proliferation, colony formation, cell migration and increased apoptosis of LR HCC cells. In vivo and in vitro results showed Sophoridine could further sensitize the therapeutic of lenvatinib against LR HCC. Mechanism studies revealed that Sophoridine decreased ETS‐1 expression to down‐regulate VEGFR2 expression along with downstream RAS/MEK/ERK axis in LR HCC cells. Hence, our study revealed that up‐regulated VEGFR2 expression could be a predicator of the resistance of lenvatinib treatment against HCC and provided a potential candidate to restore the sensitivity of lenvatinib for HCC treatment.

## 1 INTRODUCTION

Hepatocellular carcinoma (HCC) is the most common malignancy of primary liver cancer, which accounts for nearly 90% of all cases.^1^ There are multiple aetiologies responsible for HCC, including hepatitis B virus or hepatitis C virus infection, aflatoxin‐contained food consumption, alcohol abuse, obesity, type 2 diabetes and smoking.^2^, ^3^ China has a heavy burden of million‐level people with chronic hepatitis infection, leading to the incidence and mortality of HCC in China, respectively, ranks as the fourth and the third place.^4^ Globally, HCC has a population of more than 800 000 new cases in 2018, and at the same time, death cases were of more than 780 000.^5^ Moreover, men have both higher incidence and mortality rate of HCC compared to women.^5^ These data are a proxy that HCC has already become a heavy health burden, more medical investments in both preclinical and clinical practice are still in urgent need.

Currently, the curative treatment for early‐stage HCC patients includes liver resection, liver transplantation and local ablation.^6^ It is noted that most HCC patients are diagnosed with advanced stage which means they cannot undertake those treatments because of dysfunction of liver.^7^ Alternatively, only two targeted therapies sorafenib and lenvatinib have been approved by Food and Drug Administration as the standard frontline treatments for advanced HCC patients.^8^ Similar to sorafenib, lenvatinib is an orally multi‐targeted tyrosine kinase inhibitor that selectively inhibits vascular endothelial growth factor receptors (VEGFR), fibroblast growth factor receptors (FGFR), platelet‐derived growth factor receptor α (PDGFRα), KIT and RET.^9^, ^10^ It is highlighted that the resistance of targeted therapy still exists because of the primary resistance or adaptive resistance, which has hindered the treatment of advanced HCC.^11^, ^12^ Therefore, exploring the potential underlying mechanisms of lenvatinib resistance is necessary with clinical significance.

Besides the investigation of resistance mechanisms, another solution could be searching for potential combined therapy to overcome or ameliorate resistance. We have noted that Sophoridine is a natural bioactive alkaloid extracted from the seeds of *Sophora alopecuroides L* with multiple pharmacological functions,^13^ including anti‐tumour,^14^ anti‐inflammation,^15^ anti‐osteoporosis^16^ and anti‐virus.^17^, ^18^ For its anti‐tumour function, previous studies demonstrated that Sophoridine could suppress the tumour growth of gastric cancer,^13^ lung cancer,^19^ medulloblastoma,^20^ pancreatic cancer,^21^ glioma,^22^ colorectal cancer^23^ and HCC.^24^ However, the therapeutic effect of Sophoridine on lenvatinib‐resistant (LR) HCC and whether Sophoridine can sensitize HCC to lenvatinib are still unknown.

Here, we revealed that up‐regulated VEGFR2 expression and its downstream RAS/MEK/ERK signalling mediated the lenvatinib resistance of HCC. Transcription factor E26 transformation specific sequence 1 (ETS‐1) was responsible for VEGFR2 mediated lenvatinib resistance. In vivo and in vitro studies revelated Sophoridine distinctly suppressed LR HCC and sensitized the therapeutic of lenvatinib. These data provided potential evidence for the underlying mechanism of lenvatinib resistance and approved that Sophoridine could be a novel combined therapy with lenvatinib for HCC treatment.

## 2 MATERIALS AND METHODS

### 2.1 Materials

HepG2 and Huh7 human HCC cell lines were purchased from the American Type Culture Collection (ATCC). Sophoridine was obtained from Selleck (cat#S3895). Lenvatinib was bought from MCE (cat# HY‐10981). DMEM medium (cat# SH30243.01) and foetal bovine serum (cat# SH30406.05) were gained from Hyclone. Penicillin‐streptomycin (cat#15140122) and 0.25% trypsin (cat#25200072) were acquired from Gibco.

### 2.2 Cell culture

HepG2 and Huh7 cell lines were cultured in DMEM medium supplemented with 10% foetal bovine serum and 1% penicillin‐streptomycin. Then, the cell lines were maintained in cell incubator in a humidified atmosphere containing 5% CO_2_ at 37°C. For each experiment, cell lines were harvested by 0.25% trypsin.

### 2.3 Cell viability

Cell viability was measured by Cell counting kit‐8 (CCK‐8; Yeasen, cat# 40203ES60). According to the standard protocol, 5 × 10^3^ cells were seeded into 96‐well palates with three replicates. Then, cells were treated with lenvatinib or Sophoridine for 24‐96 hours at 37°C in 5% CO_2_. Last, 10 μL CCK‐8 was added into each well and incubated for another 4 hours. OD value of each well was detected by Microplate Reader at 450 nm.

### 2.4 Development of lenvatinib resistance cell lines

First, the IC_50_ of HepG2 and Huh7 cell lines to lenvatinib were detected. HepG2 or Huh7 cells were seeded into 96‐well plates and treated with various doses of lenvatinib. After incubation for 72 hours, the cell viability was determined by CCK‐8. Then, 1 × 10^4^ HepG2 or Huh7 cells were seeded into 6‐well palates and incubated with lenvatinib concentrations just below their IC_50_. During the following weeks, the dosages of lenvatinib were slowly increased at 0.25 μmol/L per time. Over 6‐7 months, we established HepG2 and Huh7 cell lines resistant to lenvatinib (HepG2‐LR and Huh7‐LR). After establishment, these resistant cell lines were continuously cultured with the presence of lenvatinib.

### 2.5 Colony formation assay

First, HepG2, HepG2‐LR, Huh7 cell and Huh7‐LR cells were seeded into 6‐well palates at a density of 500 cells/per well and then treated with lenvatinib or Sophoridine for 24 hours. Then, the drug‐contained medium was discarded, and the fresh medium was added into plates. Cells were incubated for another 2 weeks under 37°C in 5% CO_2_. Last, the colonies were fixed with 4% paraformaldehyde and stained with crystal violet.

### 2.6 Cell apoptosis assay

The apoptosis of HepG2‐LR and Huh7‐LR cells was determined by the FITC/Annexin V apoptosis detection Kit (BD Pharmingen, cat#556547). In brief, HepG2‐LR and Huh7‐LR cells were previously seeded in 6‐well plate (1 × 10^6^ cells/well) for 6 hours and then treated with different concentration of Sophoridine (0, 20, 40 and 80 µmol/L) for another 24 hours. HepG2‐LR and Huh7‐LR cells were harvested and re‐suspended in binding buffer. Next, 100 μL cell solution was transferred into 1.5 mL centrifuge tube and 5 μL FITC Annexin V and 5 μL PI was added into each tube. Finally, these tubes were incubated for 15 minutes at room temperature in the dark and 400 μL binding buffer was added into each tube before analysing by flow cytometry.

### 2.7 Cell migration assay

Cell migration was performed with 24‐transwell plates with 8 μm diameter filters. HepG2‐LR and Huh7‐LR cells were previously treated with 0, 20, 40 or 80 µmol/L Sophoridine in 6‐well plates for 24 hours. Then Sophoridine‐treated cells (1 × 10^5^) were added to the upper chamber and allowed to migrate through the filter for 12 hours. After the cells on the top of filter were removed, the cells on the bottom of the filter were fixed with 4% paraformaldehyde and stained with crystal violet. Migration cell images in the filter were collected under microscope (Nikon).

### 2.8 Quantitative real‐time PCR analysis

Following the manufacturer's instructions, total RNA from HepG2, HepG2‐LR, Huh7 and Huh7‐LR cells were collected by TRIzol reagent (Invitrogen) and RNeasy kit (Qiangen). Then, the RNA was reverse transcribed into cDNA by a FastKing One Step RT‐PCR Kit (Tiangen). qRT‐PCR was performed on 7500 real‐time PCR system (Applied Biosystems) by using SYBR green (Takara). The detailed Ct values were calculated and normalized to GAPDH. Primers were as follows: VEGFR1 forward 5′‐TTTGCCTGAAATGGTGAGTAAGG‐3′, VEGFR1 reverse 5′‐TGGTTTGCTTGAGCTGTGTTC‐3′; VEGFR2 forward 5′‐GGCCCAATAATCAGAGTGGCA‐3′, VEGFR2 reverse 5′‐CCAGTGTCATTTCCGATCACTTT‐3′; VEGFR3 forward 5′‐TGCACGAGGTACATGCCAAC‐3′, VEGFR3 reverse 5′‐GCTGCTCAAAGTCTCTCACGAA‐3′; FGFR1 forward 5′‐CCCGTAGCTCCATATTGGACA‐3′, FGFR1 reverse 5′‐ TTTGCCATTTTTCAACCAGCG‐3′; FGFR2 forward 5′‐ AGCACCATACTGGACCAACAC‐3′, FGFR2 reverse 5′‐ GGCAGCGAAACTTGACAGTG‐3′; FGFR3 forward 5′‐ TGCGTCGTGGAGAACAAGTTT‐3′, FGFR3 reverse 5′‐ GCACGGTAACGTAGGGTGTG‐3′; FGFR4 forward 5′‐ GAGGGGCCGCCTAGAGATT‐3′, FGFR4 reverse 5′‐ CAGGACGATCATGGAGCCT‐3′; PDGFRα forward 5′‐ TGGCAGTACCCCATGTCTGAA‐3′, PDGFRα reverse 5′‐ CCAAGACCGTCACAAAAAGGC‐3′; PDGFRβ forward 5′‐ AGCACCTTCGTTCTGACCTG‐3′, PDGFRβ reverse 5′‐ TATTCTCCCGTGTCTAGCCCA‐3′; ETS‐1 forward 5′‐GAGTCAACCCAGCCTATCCAGA‐3′, ETS‐1 reverse 5′‐GAGCGTCTGATAGGACTCTGTG‐3′; GAPDH forward 5′‐AAGAAGGTGGTGAAGCAGGC‐3′, GAPDH reverse 5′‐TCCACCACCCT GTTGCTGTA‐3′.

### 2.9 Western blotting

The HepG2, HepG2‐LR, Huh7 and Huh7‐LR cells were lysed in RIPA buffer. Protein concentration of each sample was detected by using BCA protein assay kit (Beyotime). Then, proteins were separated by 10% SDS‐PAGE gels and transferred to a PVDF membrane. The membrane was incubated with the indicated primary antibodies at 4°C over‐night. After incubated with HRP‐linked secondary antibody, the immunoreactive bands were detected with a chemiluminescence kit (Thermo Fisher Scientific). The primary antibodies include anti‐VEGF Receptor 2 antibody (1:1000, ab39256; Abcam), anti‐RAS antibody (1:1000, ab52939; Abcam), MEK1/2 (47E6) Rabbit antibody (1:1000, 9126; CST), Phospho‐MEK1/2 (Ser217/221) (41G9) Rabbit antibody (1:1000, 9154; CST), ERK1/2 (137F5) Rabbit antibody (1:1000, 4695; CST), Phospho‐ERK1/2 (Thr202/Tyr204) Rabbit antibody (1:1000, 4370; CST), anti‐GAPDH antibody (1:1000, ab8245; Abcam) and ETS‐1 (D8O8A) Rabbit antibody (1:1000, 14069; CST).

### 2.10 siRNA transfection

HepG2, HepG2‐LR, Huh7 and Huh7‐LR cells were transfected in 6‐well plates with either 10 nmol/L ETS‐1 siRNA (sc‐29309; Santa Cruz) or control siRNA (sc‐37007; Santa Cruz) following the general transfection protocol. After 36 hours transfection, the cells were harvested for qPCR, Western or cell viability assays.

### 2.11 In vivo study

For in vivo study, a total of 1 × 10^6^ HepG2‐LR cells were injected subcutaneously into the left flank of BALB/c nude mice (4 weeks old, male). When the tumour volumes reached around 100 mm^3^, the tumour‐bearing mice were randomized into four groups, including control group (saline solution, daily, intraperitoneally), Sophoridine group (50 mg/kg, daily, intraperitoneally), lenvatinib group (30 mg/kg, daily, intragastrically) or Sophoridine combined with lenvatinib group. Tumour volume was measured by caliper and calculated with the formula tumour volume = 1/2(length × width^2^). After 16 days, the mice were killed, and the tumour weight was also weighted and recorded.

### 2.12 Immunohistochemistry staining

HepG2‐LR tumour tissues were collected from the in vivo experiment for immunohistochemistry to detect the expression of Ki67 (ab15580; Abcam), CD31 (ab28364; Abcam), VEGFR2 (ab2349; Abcam) and p‐ERK (4370; CST) expression. In brief, paraffin‐embedded tumour tissues were dewaxed and incubated with related primary antibodies. Then, streptavidin horseradish peroxidase and 3,3’‐diaminobenzidine were added into tumour slides. Results were observed under a microscope (Nikon).

### 2.13 Statistical analysis

Data were plotted and presented as means ± SD. The difference between groups were analysed by Student's *t* tests. *P* values < .05 were considered statistically significant. *, *P* < .05; **, *P* < .01; ***, *P* < .001.

## 3 RESULTS

### 3.1 Development of lenvatinib‐resistant cell lines

**Figure 1:**
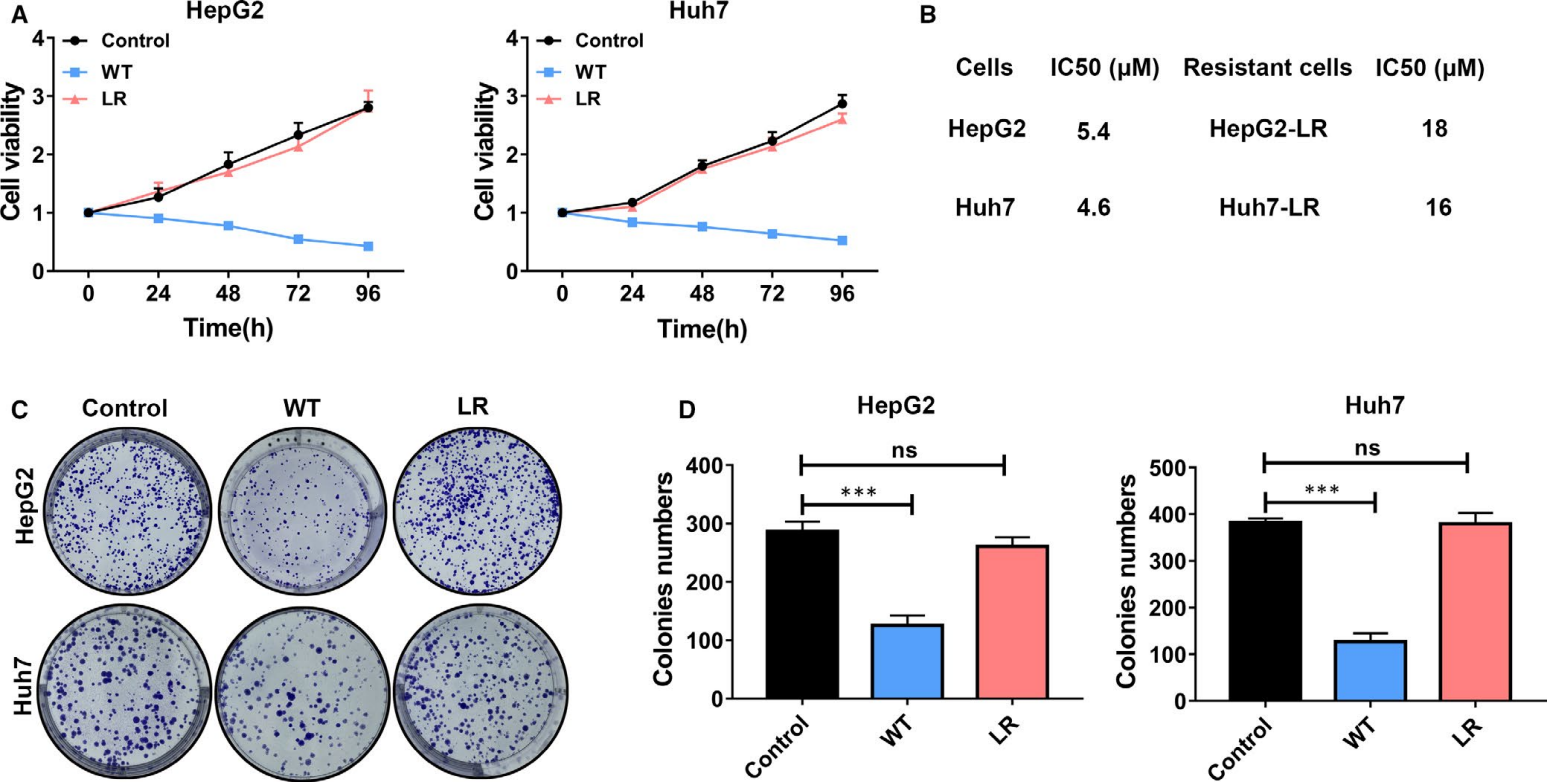
Development of lenvatinib‐resistant cell lines. A, The cell viability was measured by CCK‐8 assay at different time intervals (24, 48, 72 and 96 h) when cells were cultured with 5 μmol/L lenvatinib. The proliferation of lenvatinib‐resistant (LR) HepG2 or Huh7 cells was greater than parental HepG2 or Huh7 cells (WT). control: parental HepG2 or Huh7 cells without lenvatinib treatment; WT: parental HepG2 or Huh7 cells with lenvatinib treatment; LR: lenvatinib‐resistant HepG2 or Huh7 cells with lenvatinib treatment. B, Table with IC_50_ values of lenvatinib against the two developed resistant cell lines and their corresponding parental cells. C, Cells were exposed to 5 μmol/L lenvatinib in clonogenicity assay. Represent clone images of different indicated cells were shown. D, Quantification of the number of clones in different cells after treatment with 5 μmol/L lenvatinib. LR‐HepG2 or LR‐Huh7 cells formed more clones than their parental cells. ns, *P* value > 0.05; ***, *P* value < 0.001

In order to explore the underlying mechanism of lenvatinib resistance in HCC, we developed two LR HCC cell lines by culturing HCC cells with long‐term exposure to lenvatinib in the culture medium. LR cells were acquired by gradually increasing the dosages of lenvatinib over repeated cell passages (6‐7 months). LR cell lines were successfully constructed when HCC cell lines can tolerate higher doses of lenvatinib compared to parental cell lines. Then, two LR HepG2 and Huh7 cell lines were established (Figure 1A). Those two cell lines were characterized by higher cell viability than the parental cells (WT) with lenvatinib treatment (Figure 1A). The IC_50_ of the parental and LR cells were also determined (Figure 1B). In the resistant cells, the IC_50_ of lenvatinib showed a higher concentration compared to parental cells (Figure 1B). Accordingly, lenvatinib treatment had no influence on the growth of individual clones of resistant cells compared to their parental cells (Figure 1C,D).

### 3.2 Up‐regulated VEGFR2 expression mediated lenvatinib resistance by activating RAS/MEK/ERK signalling

**Figure 2:**
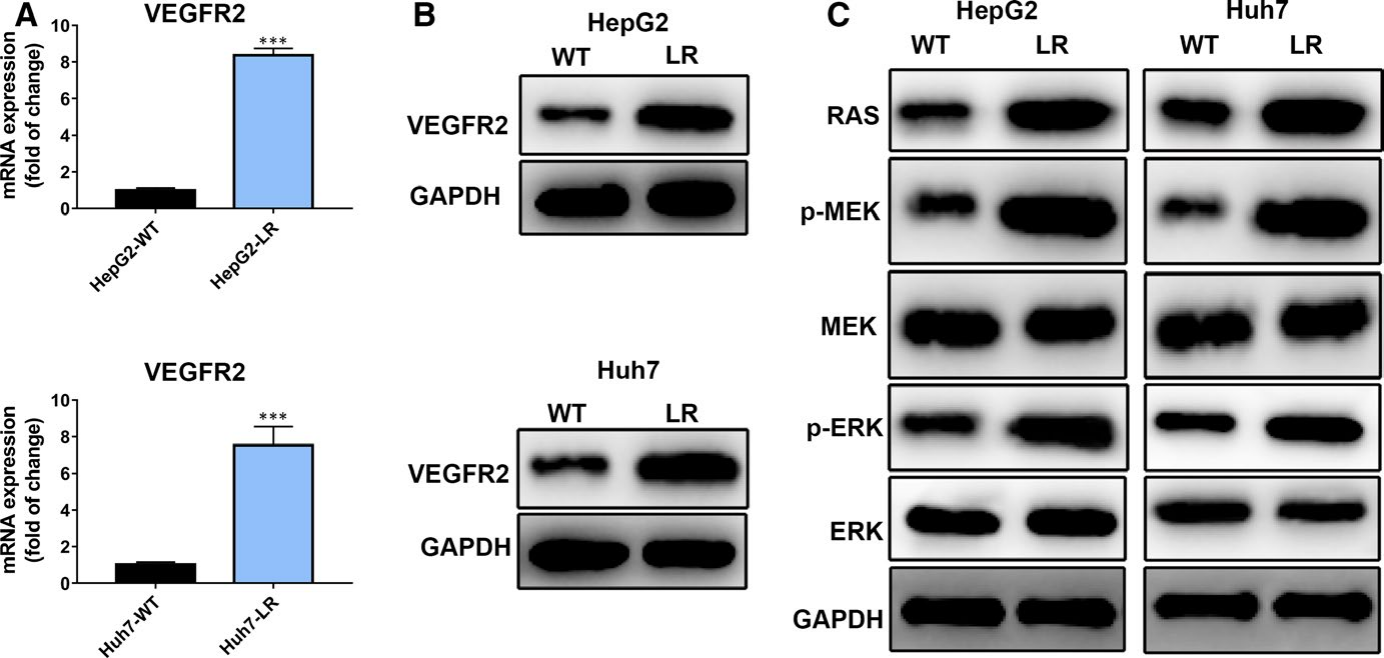
Upregulated VEGFR2 expression mediates lenvatinib resistance by activating RAS/MEK/ERK signalling. A, The mRNA expression of VEGFR2 in HepG2‐WT/LR and Huh7‐WT/LR cells. VEGFR2 mRNA expression was up‐regulated in HepG2‐LR and Huh7‐LR cells. B, The protein expression of VEGFR2 in HepG2‐WT/LR and Huh7‐WT/LR cells. C, Western blotting results showed the downstream target RAS/MEK/ERK axis of VEGFR2 were obviously up‐regulated in HepG2‐LR and Huh7‐LR cells compared with their parental cells. ***, *P* value < 0.001

As lenvatinib is multi‐targeted tyrosine kinase inhibitor including VEGFR1‐3, FGFR1‐4 and PDGFRα/β,^10^ we hereby measured the mRNA level of these cell receptors in the LR and parental cell lines by qRT‐PCR (Figure 2A and S1). qRT‐PCR assays showed VEGFR2 mRNA expression was significantly up‐regulated in HepG2‐WT/LR and Huh7‐WT/LR cells compared to parental cell lines, whereas the expression of other cell receptors had no difference between groups (Figure 2A and S1). Thus, we further measured the VEGFR2 protein levels, which also presented higher expression in LR cells (Figure 2B). RAS/MEK/ERK axis is recognized as the downstream pathway of VEGFR2.^25^ Consistently, the protein level of RAS was increased and the p‐MEK, p‐ERK levels were also up‐regulated in LR cells compared to parental cells (Figure 2C). These results suggested that up‐regulated VEGFR2 expression may mediate lenvatinib resistance by activating RAS/MEK/ERK signalling.

### 3.3 ETS‐1 was responsible for VEGFR2 mediated lenvatinib resistance

**Figure 3:**
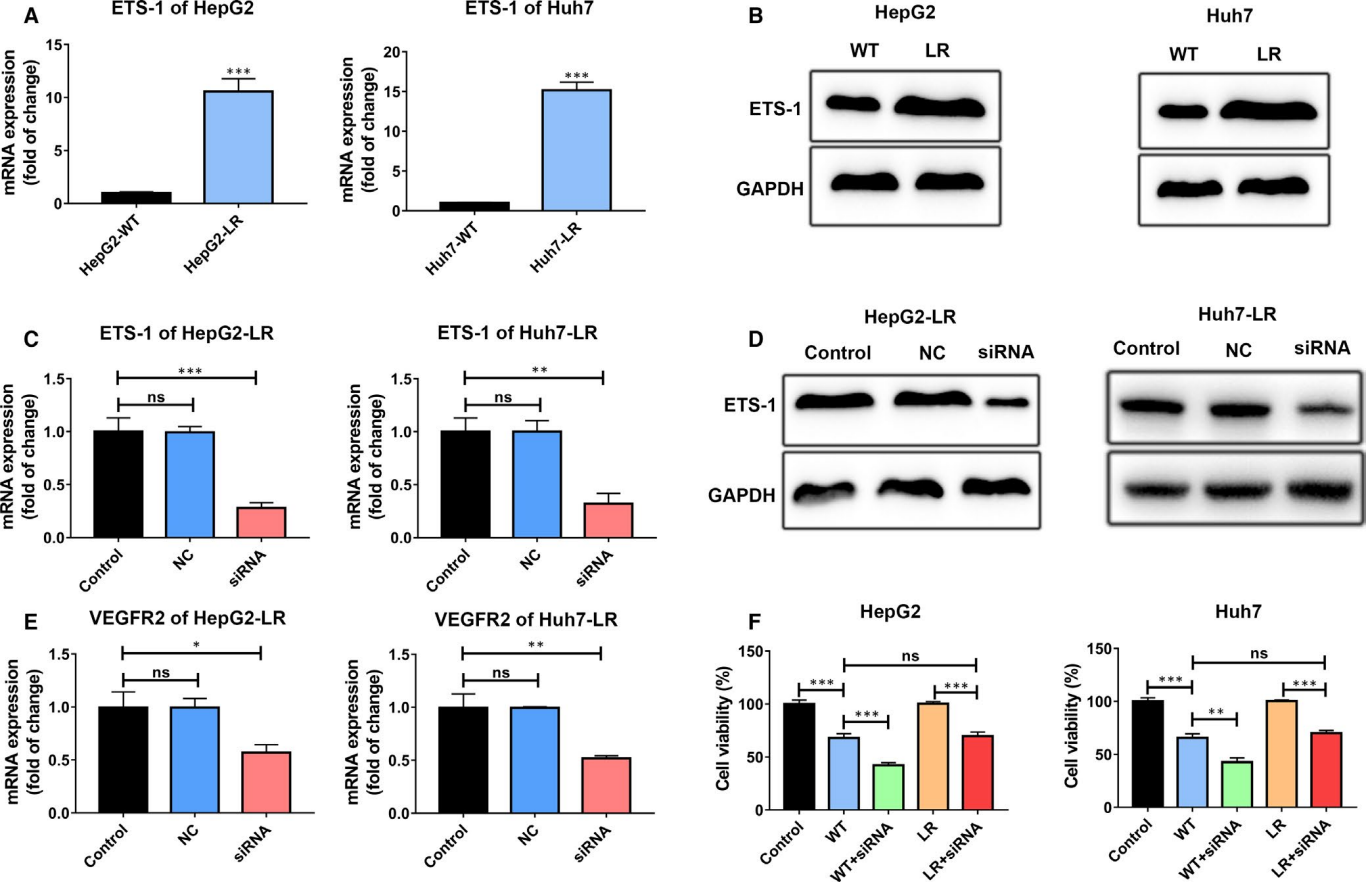
ETS‐1 was responsible for VEGFR2 mediated lenvatinib resistance. A, The mRNA expression of ETS‐1 in HepG2‐WT/LR and Huh7‐WT/LR cells. ETS‐1 mRNA expression was increased in HepG2‐LR and Huh7‐LR cells. B, The protein expression of ETS‐1 in HepG2‐WT/LR and Huh7‐WT/LR cells. C and D, HepG2‐LR and Huh7‐LR cells were transfected with control siRNA (NC) or ETS‐1 siRNA. The knock down efficiency of ETS‐1 siRNA was detected. The mRNA and protein expression of ETS‐1 were quantified by qPCR and Western blotting. E, The mRNA expression of VEGFR2 after knocking down ETS‐1 expression in HepG2‐LR and Huh7‐LR cells. F, The indicated cells were treated with 5 μmol/L lenvatinib for 48 h, and then, cell viability was detected by CCK‐8 assay. ETS‐1 knock down rescued the efficacy of lenvatinib against lenvatinib‐resistant HCC cells. Control: parental HepG2 or Huh7 cells; WT: parental HepG2 or Huh7 cells with lenvatinib treatment; WT + siRNA: parental HepG2 or Huh7 cells were firstly transfected with ETS‐1 siRNA and then treated with lenvatinib; LR: lenvatinib‐resistant HepG2 or Huh7 cells with lenvatinib treatment; LR + siRNA: lenvatinib‐resistant HepG2 or Huh7 cells were firstly transfected with ETS‐1 siRNA and then treated with lenvatinib. ns, *P* value > 0.05; *, *P* value < 0.05; **, *P* value < 0.01; ***, *P* value < 0.001

Previous studies have pointed out that activation of ETS‐1 promotes the FOX:ETS motif bind to the first intron enhancer of VEGFR2 to enhance VEGFR2 expression.^26^ To further explore the underlying mechanism of regulating VEGFR2 expression in LR HCC cells, we detected mRNA and protein expression of ETS‐1 in HepG2‐WT/LR and Huh7‐WT/LR cells (Figure 3A,B). Results showed ETS‐1 mRNA and protein expression were obviously enhanced in HepG2‐LR and Huh7‐LR cells compared to their parental cells (Figure 3A,B). Next, we wondered whether ETS‐1 down‐regulation could decrease the expression of VEGFR2 and rescue the suppressive effect of lenvatinib on HepG2‐LR and Huh7‐LR cells. ETS‐1 siRNA was used to knockdown the expression of ETS‐1 in HepG2‐WT/LR and Huh7‐WT/LR cells. The knockdown efficiency of ETS‐1 siRNA was evaluated by qPCR and Western blotting (Figure 3C,D). ETS‐1 siRNA effectively decreased the mRNA and protein expression of ETS‐1 in HepG2‐LR and Huh7‐LR cells. After knocking down ETS‐1 expression, the mRNA expression of VEGFR2 was also inhibited, which justified the role of ETS‐1 in regulating VEGFR2 expression (Figure 3E). We further explored the influence of lenvatinib on the cell viability of HepG2‐WT/LR and Huh7‐WT/LR cells after knocking down ETS‐1. Results showed knocking down ETS‐1 reversed the sensitivity of HepG2‐LR or Huh7‐LR cells to lenvatinib compared to HepG2‐WT or Huh7‐WT cells (Figure 3F). Herein, we concluded that ETS‐1 was responsible for VEGFR2 mediated lenvatinib resistance.

### 3.4 Sophoridine inhibited the proliferation, colony formation and increased apoptosis of lenvatinib‐resistant HCC cells

**Figure 4:**
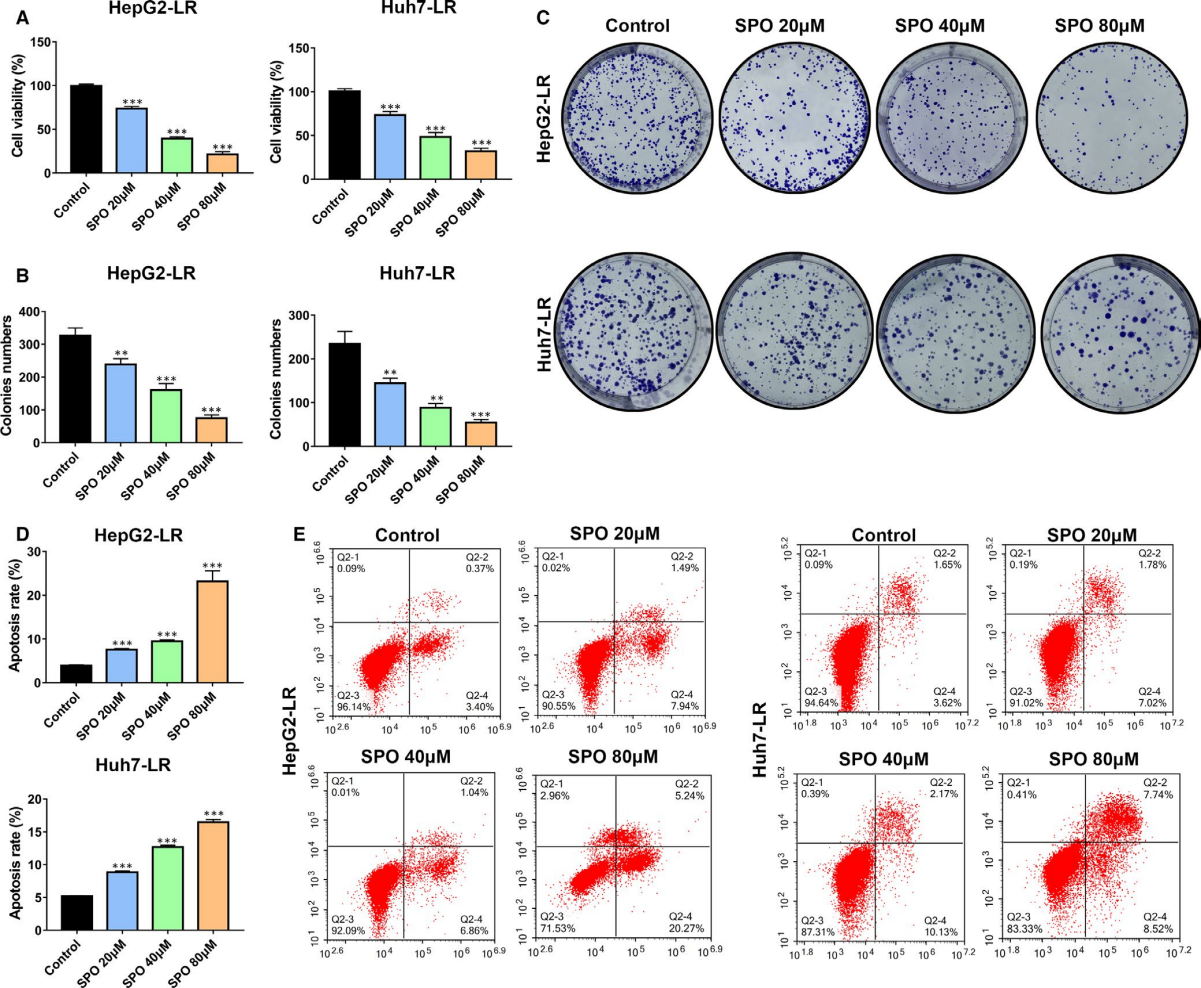
Sophoridine inhibited the proliferation, colony formation and increased apoptosis of lenvatinib‐resistant HCC cells. A, Lenvatinib‐resistant (LR) HepG2 or Huh7 cells were treated with different doses (20, 40 and 80 μmol/L) of Sophoridine (SPO) for 72 h. The viability of HepG2‐LR and Huh7‐LR cells was determined by CCK‐8 assay. Sophoridine treatment suppressed the growth of HepG2‐LR and Huh7‐LR cells. B, The HepG2‐LR and Huh7‐LR cells were treated with 20, 40 or 80 μmol/L Sophoridine in colony formation assay. The colonies formation of HepG2‐LR and Huh7‐LR cells were inhibited after Sophoridine treatment. C, Represent clone images were shown in different groups after indicated treatments. D, The apoptosis of HepG2‐LR and Huh7‐LR cells were detected by the Annexin‐V/PI staining assay through flow cytometry after 20, 40 or 80 μmol/L Sophoridine treatment for 24 h. Sophoridine induced the apoptosis of HepG2‐LR and Huh7‐LR cells. E, The representative gating images of Sophoridine on the HepG2‐LR or Huh7‐LR cells apoptosis were shown. **, *P* value < 0.01; ***, *P* value < 0.001

To explore whether Sophoridine treatment could influence the proliferation, colony formation and apoptosis of LR HCC cells, HepG2‐LR and Huh7‐LR cells were treated with 20, 40 and 80 μmol/L Sophoridine. Cell viability was determined by CCK‐8 assay, and results showed Sophoridine obviously suppressed the growth of HepG2‐LR and Huh7‐LR cells in dose‐dependent manner (Figure 4A). In colony formation assays, Sophoridine significantly inhibited the growth of individual clones of HepG2‐LR and Huh7‐LR cells (Figure 4B,C). At the same time, flow cytometry results also showed Sophoridine induced the apoptosis of LR cells in a dose‐dependent manner (Figure 4D,E). Thus, these results suggested that Sophoridine inhibited the proliferation, colony formation and increased apoptosis of LR HCC cells.

### 3.5 Sophoridine suppressed the migration of lenvatinib‐resistant HepG2 and Huh7 cells

**Figure 5:**
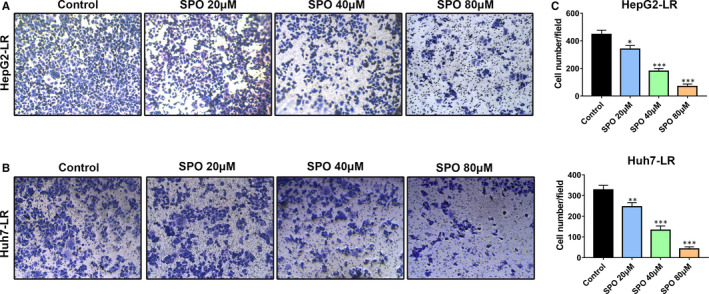
Sophoridine suppressed the migration of lenvatinib‐resistant HepG2 and Huh7 cells. A, B, Lenvatinib‐resistant (LR) HepG2 or Huh7 cells were previously treated with 20, 40 or 80 μmol/L of Sophoridine for 24 h. Representative images of the migration ability of HepG2‐LR and Huh7‐LR cells were shown. C, The numbers of migrated HepG2‐LR and Huh7‐LR cells were quantified. *, *P* value < 0.05; **, *P* value < 0.01; ***, *P* value < 0.001

To further investigate the influence of Sophoridine on the migration ability of LR HCC cells, HepG2‐LR (Figure 5A) and Huh7‐LR cells (Figure 5B) were previously treated with 20, 40 or 80 μmol/L of Sophoridine for 24 hours. Then, the migration of Sophoridine‐treated HCC cells was assessed by 24‐well‐tranwell assay. Results showed Sophoridine could suppressed the migration of HepG2‐LR and Huh7‐LR cells in a dose‐dependent manner (Figure 5C).

### 3.6 Sophoridine sensitized the anti‐tumour effect of lenvatinib against lenvatinib‐resistant HCC cell lines in vitro and in vivo

**Figure 6:**
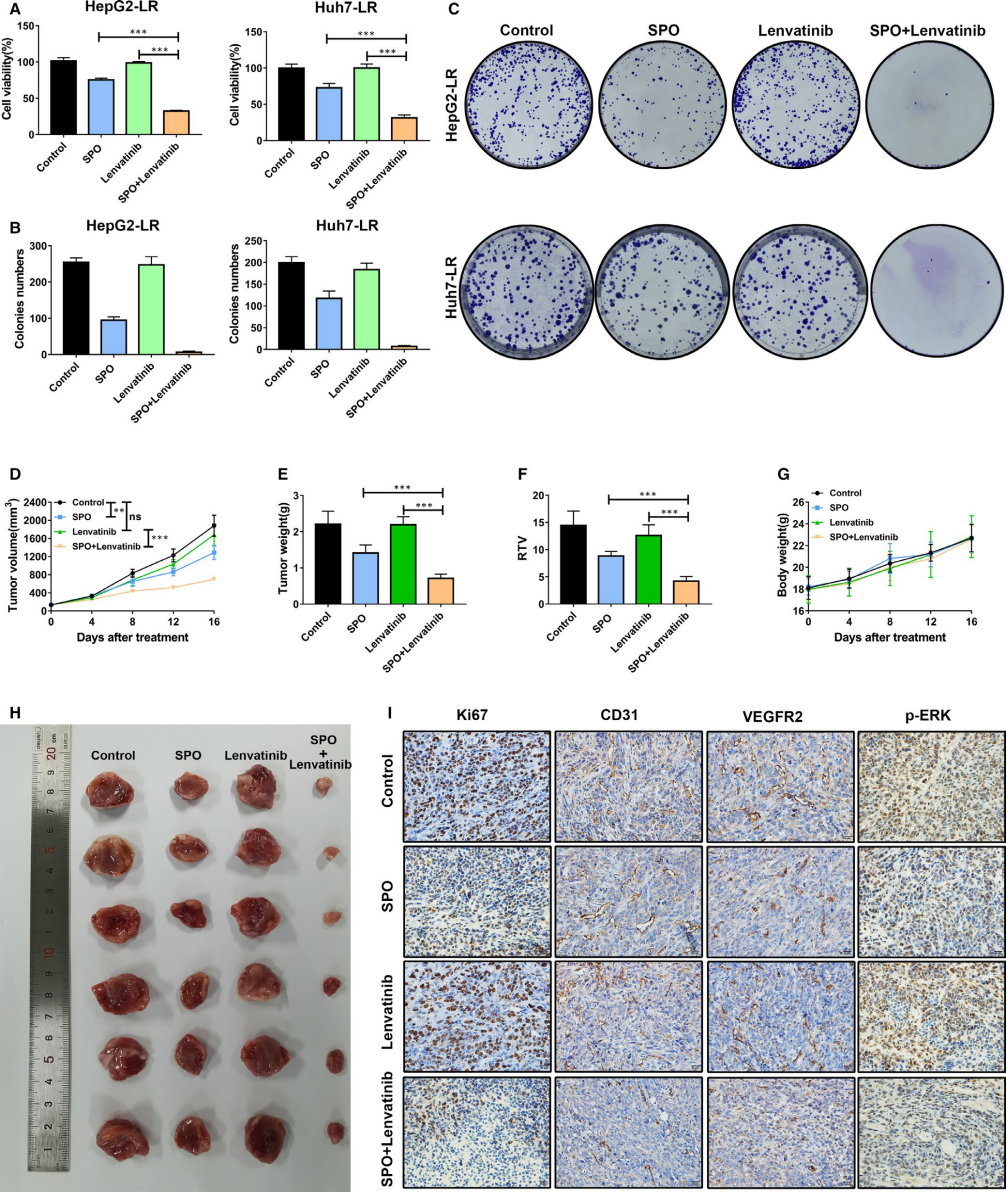
Sophoridine sensitized the anti‐tumour effect of lenvatinib against lenvatinib‐resistant HCC cell lines in vitro and in vivo. A, Lenvatinib‐resistant (LR) HepG2 or Huh7 cells were treated with 20 μmol/L Sophoridine combined with 5 μmol/L lenvatinib for 72 h. The combination treatment showed the most effective influence on the growth of HepG2‐LR and Huh7‐LR cells. B, The HepG2‐LR and Huh7‐LR cells were treated with 20 μmol/L Sophoridine combined with 5 μmol/L lenvatinib in colony formation assay. Colonies formation of HepG2‐LR and Huh7‐LR cells were inhibited after combination treatment. C, Representative images of the colony formation ability of HepG2‐LR and Huh7‐LR cells were shown after indicated treatments. D, BALB/c nude mice were subcutaneously burdened with HepG2‐LR cells. Mice were divided into different treatment groups after 7 days (n = 6). Mice were treated with Sophoridine (50 mg/kg, daily, intraperitoneally), lenvatinib (30 mg/kg, daily, intragastrically) or Sophoridine combined with lenvatinib. Tumour growth change curves were explored after indicated treatment. E, Tumour weight was detected after mice killing. F, The relative tumour volume (RTV) ratio was calculated according to the formula RTV = tumour volume day 16‐day 0/tumour volume day 0. G, Mouse bodyweight changes were observed during the treatments. H, The representative photograph of tumours was shown in different groups. I, Tumour slices of indicated groups were stained with Ki67, CD31, VEGFR2 and p‐ERK. The representative immunohistochemistry images of Ki67, CD31, VEGFR2 and p‐ERK from different groups were displayed. ns, *P* value > 0.05; **, *P* value < 0.01; ***, *P* value < 0.001

Because of Sophoridine had a distinct effect on the growth of LR HCC cells, we then proposed whether Sophoridine synergized with lenvatinib could have a more efficient suppression influence on LR HCC cells than alone treatment. We first assessed the cell viability and colony formation ability of LR HCC cells with Sophoridine, lenvatinib or combination treatment in vitro (Figure 6A‐C). The combination treatment exerted the most effective suppression effect on cell viability compared to alone treatment (Figure 6A). Accordingly, Sophoridine combined with lenvatinib also furthest inhibited the colony formation of HepG2‐LR and Huh7‐LR cells (Figure 6B,C).

We also established HepG2‐LR cell subcutaneous tumour model in BALB/c nude mice to perform in vivo study. The tumour‐burdened mice were randomly divided into control, Sophoridine, lenvatinib and Sophoridine combined with lenvatinib groups. Tumour volumes and mice bodyweight variation were recorded during all treatments (Figure 6D,G). At the end of treatment, those mice were killed, and tumour tissues were weighted and pictured (Figure 6E,H). Moreover, we further calculated the relative tumour volume (RTV) ratio (Figure 6F). Results showed lenvatinib treatment had no influence on mice tumour volumes, tumour weight and RTV ratio, which was consistent with in vitro results that established HepG2‐LR cells was resistant to lenvatinib (Figure 6D‐F). Sophoridine combined with lenvatinib showed the most effective suppression influence on the mice tumour volumes, tumour weight and RTV ratio compared to Sophoridine treatment alone (Figure 6D‐F). Meanwhile, the combination treatment had no influence on the mice bodyweight, indicating the safety of treatment (Figure 6G). To further assess the effect of Sophoridine treatment on the tumour cell proliferation and angiogenesis, we performed Ki67 and CD31 staining (Figure 6I). Sophoridine treatment alone suppressed the expression of Ki67 and CD31 expression and combination treatment showed the most suppressive effect, which indicated that Sophoridine treatment inhibited the tumour cell proliferation and angiogenesis in vivo. Hence, we concluded that Sophoridine sensitized the anti‐tumour effect of lenvatinib against LR HCC.

### 3.7 Sophoridine decreased ETS‐1 expression to down‐regulate VEGFR2 expression along with downstream RAS/MEK/ERK axis in lenvatinib‐resistant HCC cells

**Figure 7:**
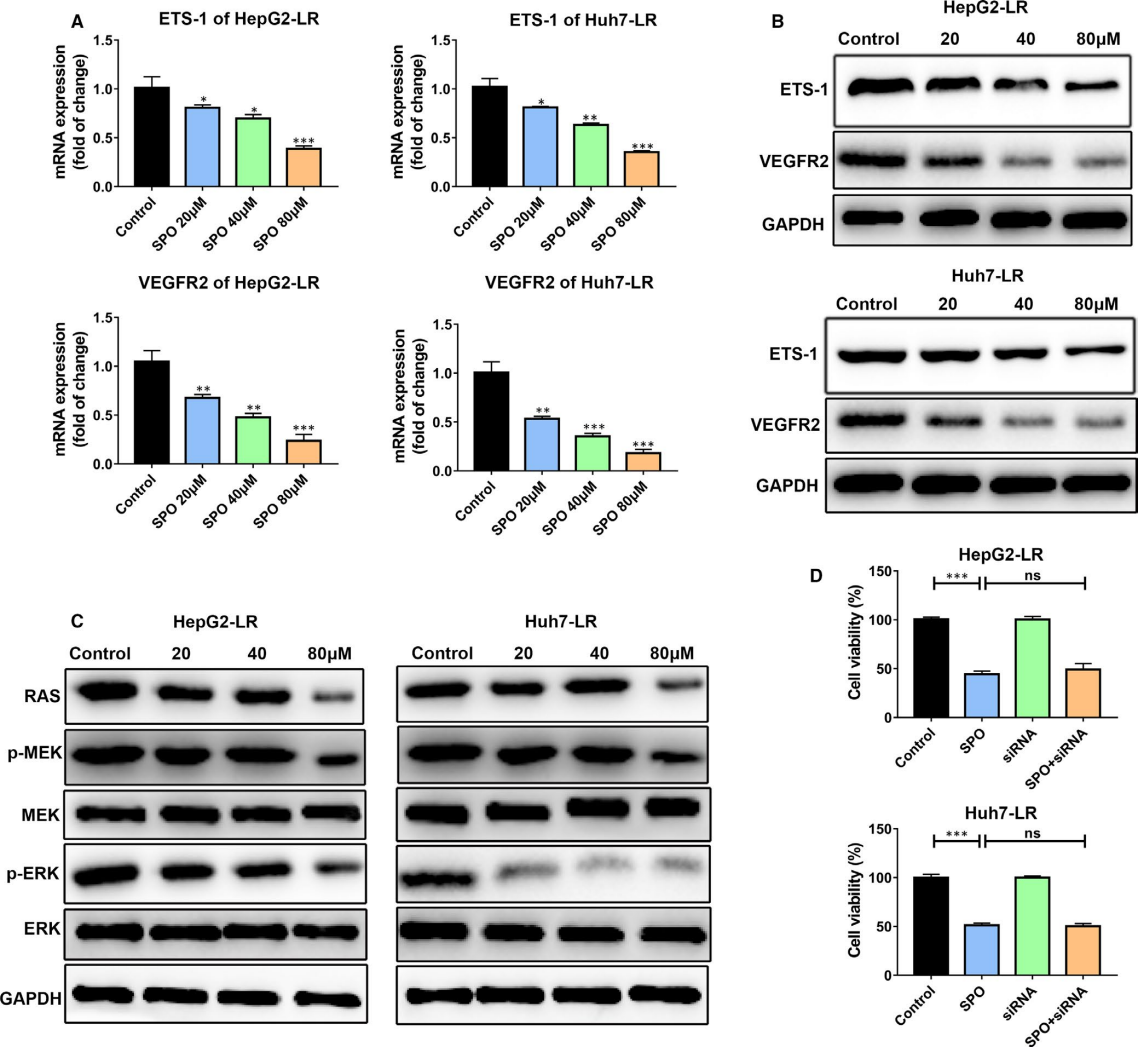
Sophoridine down‐regulated VEGFR2 expression along with downstream RAS/MEK/ERK axis in lenvatinib‐resistant HCC cells. A, Lenvatinib‐resistant (LR) HepG2 or Huh7 cells were treated with different doses of Sophoridine for 24 h. Sophoridine treatment down‐regulated ETS‐1 and VEGFR2 mRNA expression in HepG2‐LR and Huh7‐LR cells. B, The protein expression of ETS‐1 and VEGFR2 was detected by Western blotting in HepG2‐LR and Huh7‐LR cells after Sophoridine treatment. C, The downstream RAS/MEK/ERK axis was significantly inhibited in Sophoridine‐treated HepG2‐LR and Huh7‐LR cells. D, The indicated cells were treated with 40 μmol/L Sophoridine for 72 h, and then, the cell viability was detected by CCK‐8 assay. Control: HepG2‐LR or Huh7‐LR cells; SPO: HepG2‐LR or Huh7‐LR cells with Sophoridine treatment; siRNA: HepG2‐LR or Huh7‐LR cells were transfected with ETS‐1 siRNA; SPO + siRNA: HepG2‐LR or Huh7‐LR cells were firstly transfected with ETS‐1 siRNA and then treated with Sophoridine. ns, *P* value > 0.05; *, *P* value < 0.05; **, *P* value < 0.01; ***, *P* value < 0.001

We have demonstrated that up‐regulated VEGFR2 expression mediated lenvatinib resistance by activating downstream RAS/MEK/ERK signalling in vitro. We also would like to know whether Sophoridine could influence the VEGFR2 expression and subsequently inhibited the RAS/MEK/ERK axis activation to play an anti‐tumour effect against LR HCC cells. First, we measured the expression of VEGFR2 and p‐ERK in tumours after indicated treatment by immunohistochemistry (Figure 6I). Results showed that Sophoridine treatment decreased the VEGFR2 and p‐ERK expression in tumours compared to control group (Figure 6I). Then, we further explored the influence of Sophoridine on HepG2‐LR or Huh7‐LR cells in vitro (Figure 7). HepG2‐LR or Huh7‐LR cells were treated with different doses of Sophoridine for 24 hours. The mRNA and protein expression of ETS‐1 and VEGFR2 in HepG2‐LR and Huh7‐LR cells were determined by qRT‐PCR and Western blotting assays (Figure 7A,B). Results showed the mRNA expression of ETS‐1 and VEGFR2 were significantly down‐regulated after Sophoridine treatment with a dose‐dependent manner (Figure 7A). Consistently, Sophoridine also decreased the ETS‐1 and VEGFR2 protein levels in HepG2‐LR and Huh7‐LR cells (Figure 7B). Levels of RAS, p‐MEK, MEK, p‐ERK and ERK were further quantified. Western blotting results showed Sophoridine reduced the RAS, p‐MEK and p‐ERK levels, suggesting the activation of downstream RAS/MEK/ERK axis of VEGFR2 was inhibited (Figure 7C). To further explore whether Sophoridine act on ETS‐1 to regulate VEGFR2 and its downstream RAS/MEK/ERK axis expression, we firstly knocked down ETS‐1 expression in HepG2‐LR or Huh7‐LR cells. Then, HepG2‐LR or Huh7‐LR cells were further treated with Sophoridine. The cell viability results showed Sophoridine exerted a similar suppressive effect on ETS‐1‐knocked‐down HepG2‐LR or Huh7‐LR cells than HepG2‐LR or Huh7‐LR cells. Hence, Sophoridine decreased ETS‐1 expression to down‐regulate VEGFR2 expression along with downstream RAS/MEK/ERK axis in LR HCC cells, which explained the underlying mechanism responsible for increasing the sensitivity of LR HCC to lenvatinib treatment.

## 4 DISCUSSION

Hepatocellular carcinoma is hard to discern at the early stage based on regular body check, leading to the high occurrence of advanced HCC.^27^ With the lack of effective therapies, 5‐year survival of HCC patients is only 18%.^3^, ^28^ Sorafenib was the first approved targeted therapy; however, the clinical benefits of sorafenib were modest and the 5‐year relative survival remains low.^8^, ^29^ The clinical trials of lenvatinib showed lenvatinib had a significant clinical improvement in objective response rate, time to progression and progression‐free survival compared to sorafenib.^30^ Hence, the discovery and approvement of lenvatinib is an important breakthrough for advanced HCC patients. However, advanced HCC patients may develop lenvatinib resistance after a period of treatment.^11^ Similar with sorafenib, drug resistance of lenvatinib treatment remained as a major concern for such targeted therapy. In order to elucidate the molecular basis for acquired lenvatinib resistance, we developed two human HCC cell lines in which resistance to lenvatinib was acquired by continuous exposure to lenvatinib. Based on the cell models, we further explored the potential strategies for restoration of sensitivity of lenvatinib. Key findings included the following: (a) we identified that VEGFR2 overexpression and downstream RAS/MEK/ERK pathway activation had a close link with lenvatinib resistance in HCC; (b) ETS‐1 was responsible for VEGFR2 mediated lenvatinib resistance; (c) Sophoridine distinctly suppressed the growth and induced the apoptosis of LR HCC, further sensitized the effect of lenvatinib in the vitro/vivo studies; and (d) Sophoridine reduced ETS‐1 expression to down‐regulate VEGFR2 expression along with downstream RAS/MEK/ERK axis in LR HCC cells, indicating its potential as suitable candidate to combine with lenvatinib for HCC patients treatment.

Up‐regulated VEGFR expression is recognized as a critical pathway in the development and progression in the majority of solid tumours.^31^ VEGF/VEGFR axis promotes tumour angiogenesis, tumour cell proliferation and tumour metastasis through paracrine and autocrine signalling. In HCC, previous studies found VEGFR and VEGF could be simultaneously overexpressed in several human HCC cell lines to promote cell proliferation and invasiveness and inhibit apoptosis, such as 7721, 7402, HepG2, MHCC‐97H and Huh7.^31^, ^32^, ^33^ VEGFRs family include VEGFR1, VEGFR2 and VEGFR‐3 in which high expression of VEGFR2 is found in patients with chronic hepatitis B infection and is associated with the progression and prognosis of HCC.^34^ Moreover, VEGFR2 can be used as an biomarker for the therapeutic effect of sorafenib against HCC.^32^ Hence, targeting VEGFR2 can be a strategy for HCC treatment. Here, we found VEGFR2 was significantly up‐regulated during the establishment of lenvatinib resistance in HepG2 and Huh7 cells, indicating VEGFR2 could also be a predicator of the efficacy of lenvatinib against HCC. Moreover, we discovered Sophoridine showed an effective inhibition of VEGFR2 expression, which provided a potential drug candidate targeting VEGFR2.

RAS/MEK/ERK axis is recognized as the downstream pathway of VEGFR2.^25^ Ras is the first intracellular effector of MEK/ERK pathway, and ERK is the main substrate of MEK.^6^ The highest level of Ras effectors is characterized by a short survival of human HCC, indicating RAS pathway can be a prognostic implication for HCC.^6^ In HCC, the activation of MEK/ERK pathway is supportive for tumour progression by promoting cellular proliferation and survival, tumour growth, cell motility, invasiveness and angiogenesis.^35^ The RAS/MEK/ERK pathway is activated in 50%‐100% of HCC and has a correction with a poor prognosis.^6^ Hence, inhibiting RAS/MEK/ERK axis activation is vital in suppressing HCC progression. Here, we found the up‐regulated VEGFR2 expression distinctly activated RAS/MEK/ERK signalling in LR HepG2 and Huh7 cells, which confirmed the supportive role of RAS/MEK/ERK axis in HCC.

Previously, researches showed Sophoridine could inhibit colorectal carcinoma, gastric cancer, glioma, lung cancer, medulloblastoma, pancreatic cancer through inhibiting the activity of ubiquitin‐proteasome, remodelling tumour‐associated macrophages polarization via TLR4 pathway, up‐regulating caspases and PARP expression to induce cell apoptosis, activating the p53 and Hippo signalling pathways, and inducing cell cycle arrest in G0/G1 phase or S phase arrest.^13^, ^19^, ^20^, ^21^, ^22^ However, the effect and underlying mechanism of Sophoridine against LR HCC is unknown. In this research, we found Sophoridine inhibited the proliferation, colony formation, increased apoptosis and suppressed the migration of LR HCC cells in vitro. Further, Sophoridine sensitizes the anti‐tumour effect of lenvatinib against LR HCC cell lines in vitro and in vivo. Mechanism studies revealed that Sophoridine decreased ETS‐1 expression to down‐regulate VEGFR2 expression along with downstream RAS/MEK/ERK axis in LR HCC cells.

## 5 CONCLUSION

In summary, we identified the responsible role of ETS‐1 induced increased VEGFR2 expression and its downstream RAS/MEK/ERK axis activation in LR HCC for the first time. In addition, we found a novel function and mechanism of Sophoridine against LR HCC. Sophoridine had the capacity to restore the sensitivity of lenvatinib against LR HCC via suppressing ETS‐1 mediated up‐regulated VEGFR2 expression. Hence, we uncovered the underlying mechanism of lenvatinib resistance in HCC and provided an alternative candidate for sensitizing the therapeutic effect of lenvatinib against LR HCC.

## CONFLICT OF INTEREST

The authors confirm that there are no conflicts of interest.

## AUTHOR CONTRIBUTIONS

**Zhongwei Zhao:** Conceptualization (equal); Data curation (equal); Formal analysis (equal); Investigation (lead); Methodology (equal); Project administration (equal); Resources (equal); Writing‐original draft (lead); Writing‐review & editing (equal). **Dengke Zhang:** Conceptualization (equal); Data curation (equal); Formal analysis (equal); Investigation (equal); Methodology (equal); Project administration (equal); Resources (equal); Writing‐original draft (equal); Writing‐review & editing (equal). **Wu Fazong:** Data curation (equal). **Janfei Tu:** Data curation (equal). **Jingjing Song:** Investigation (equal). **Min Xu:** Funding acquisition (equal); Supervision (equal); Writing‐review & editing (equal). **Jiansong Ji:** Conceptualization (lead); Funding acquisition (lead); Supervision (lead); Writing‐original draft (equal); Writing‐review & editing (equal).

## Supporting information

Fig S1

## Data Availability

All data generated or analysed during this study are included in this article.
