# Supplementary figures and images for: Sophoridine suppresses lenvatinib‐resistant hepatocellular carcinoma growth by inhibiting RAS/MEK/ERK axis via decreasing VEGFR2 expression
Source: J Cell Mol Med. 2020 Nov 18;25(1):549–60. doi: 10.1111/jcmm.16108 (PMC7810959; doi:10.1111/jcmm.16108)

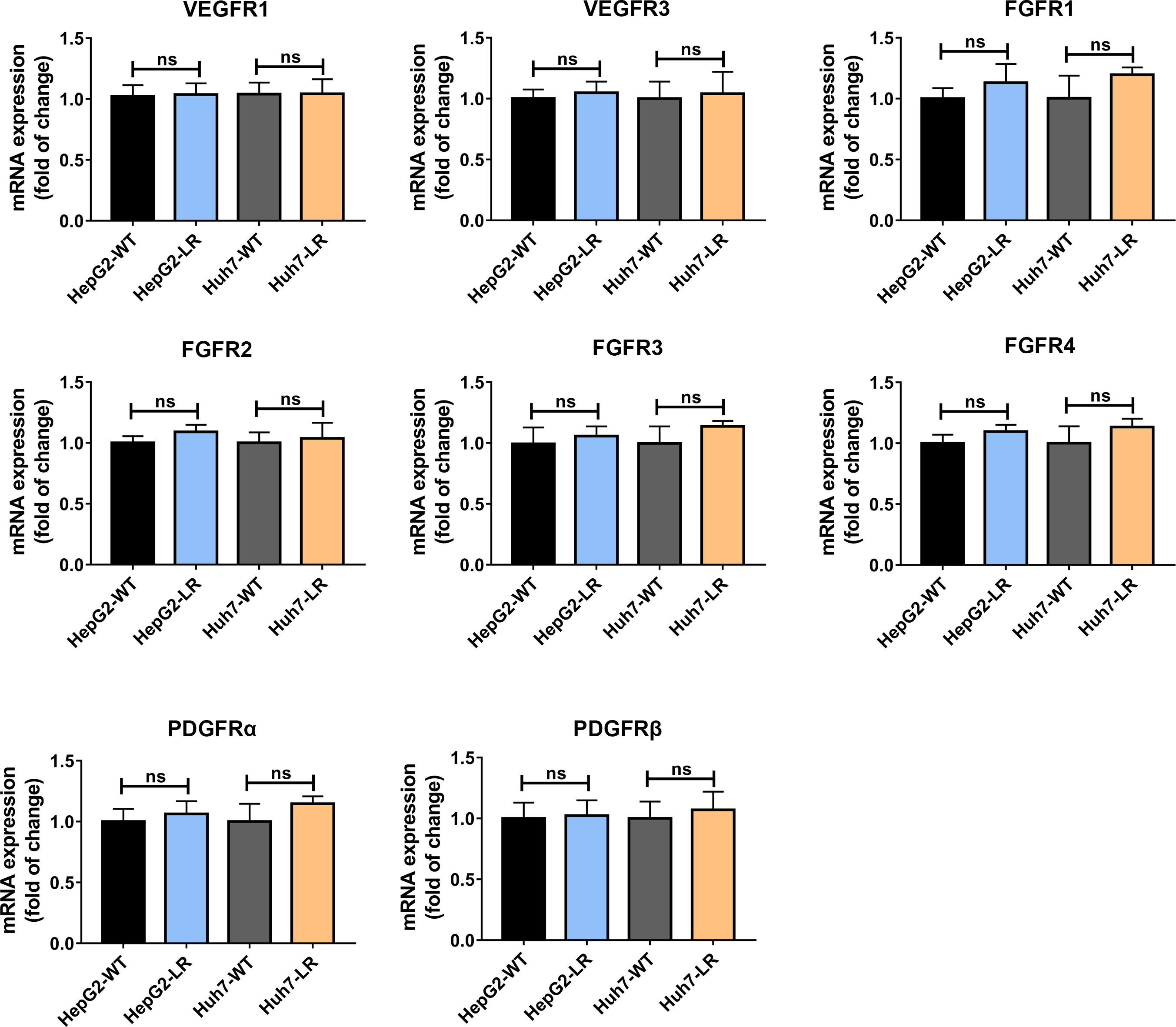

Supplement: Supplementary file 1 — Fig S1 [file JCMM-25-549-s001.tif]
